# Acute Phase CD8+ T Lymphocytes against Alternate Reading Frame Epitopes Select for Rapid Viral Escape during SIV Infection

**DOI:** 10.1371/journal.pone.0061383

**Published:** 2013-05-06

**Authors:** Andrew D. Walsh, Benjamin N. Bimber, Arpita Das, Shari M. Piaskowski, Eva G. Rakasz, Alexander T. Bean, Philip A. Mudd, Adam J. Ericsen, Nancy A. Wilson, Austin L. Hughes, David H. O'Connor, Nicholas J. Maness

**Affiliations:** 1 Department of Pathology and Laboratory Medicine, University of Wisconsin-Madison, Madison, Wisconsin, United States of America; 2 Oregon National Primate Research Center, Oregon Health and Science University, Beaverton, Oregon, United States of America; 3 Division of Microbiology, Tulane National Primate Research Center, Covington, Louisiana, United States of America; 4 Wisconsin National Primate Research Center, University of Wisconsin-Madison, Madison, Wisconsin, United States of America; 5 Medical Scientist Training Program, University of Wisconsin-Madison, Madison, Wisconsin, United States of America; 6 Department of Biological Sciences, University of South Carolina, Columbia, South Carolina, United States of America; 7 Department of Microbiology and Immunology, Tulane University School of Medicine, New Orleans, Louisiana, United States of America; University of Pittsburgh Center for Vaccine Research, United States of America

## Abstract

CD8+ T Lymphocytes (CTL) can control AIDS virus replication. However, natural selection favoring viral variants that escape CTL recognition is a common feature of both simian immunodeficiency virus (SIV) infection of macaques and HIV infection of humans. Emerging data indicate that CTL directed against alternate reading frame (ARF)-derived epitopes (a.k.a. cryptic epitopes) are important components of the total virus-specific response in SIV and HIV infection but the contributions of these responses during the critical first several weeks of infection have not been determined. We used a focused deep sequencing approach to examine acute phase viral evolution in response to CTL targeting two polypeptides encoded by ARFs of SIVmac239 in SIV-infected rhesus macaques. We report high magnitude CTL responses as early as three weeks post-infection against epitopes within both ARFs, which both overlap the 5′ end of the *env* gene. Further, mutations accumulated in the epitopes by three to four weeks post infection consistent with viral escape. Interestingly, these mutations largely maintained the primary amino acid sequence of the overlapping Envelope protein. Our data show that high frequency CTL target cryptic epitopes and exert selective pressure on SIV during the acute phase, underscoring the importance of these unique immune responses.

## Introduction

The importance of CD8+ T Lymphocytes (CTL) in controlling AIDS virus replication is well established by several lines of evidence. First, experimental depletion of CD8+ cells in rhesus macaques leads to a loss of viral control, both in the acute phase [1], [2], and during the chronic phase in animals exhibiting extraordinary control of SIV replication, elite controllers (ECs) [3]. Additionally, ECs are significantly enriched for particular MHC-I alleles in both HIV-infected humans [4]–[7] and SIV-infected macaques [8], [9], implicating a causative role for CTL in viral control. Finally, CTL select for viral escape variants in HIV and SIV during both the acute and chronic phases of infection [10]–[14]. In fact, CTL are major drivers of viral evolution [10], [11], substantiating the important interactions between CTL and virus-derived peptides presented by MHC-I molecules on the surface of infected cells.

Recent data indicate that viral alternate reading frames (ARFs) might encode important CTL targets [15]–[20]. CTL directed against an ARF-encoded epitope (a.k.a. cryptic epitope) presented by the macaque MHC-I molecule Mamu-B*017:01 selected for viral escape in SIVmac239-infected macaques and effectively suppressed SIV replication *in vitro* [18]. Indeed, SIVmac239 encodes epitopes derived from at least eight viral ARFs [21]. Furthermore, CTL recognizing these epitopes can be high frequency and can comprise up to 25% of the total anti-SIV responses in infected macaques [21]. Similar phenomena have been described in HIV-1-infected humans [17], [19], [20], [22]–[24], including during acute infection [19], [22].

**Figure 1:**
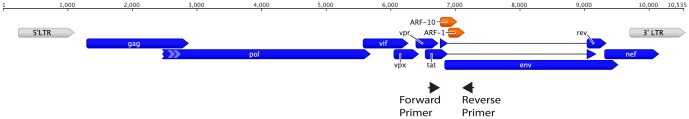
The genomic locations of ARFs -1 and -10 as well as the primers used to amplify this region for pyrosequencing, within the SIVmac239 genome. The SIVmac239 genome is 10,535 nt in length. The ORFs encoding the classical viral proteins are shown in blue and the ORFs encoding the ARFs studied in this report are shown in orange. Note that ARF-10 is depicted as separate from Rev exon 1 to emphasize its independent translation. However, ARF-10 is a composite of the first exon of Rev and the first 50 amino acids translated from the Rev intron. The relative locations of the forward and reverse primers used to amplify viral RNA for pyrosequencing are shown as labeled black arrows. This image was created using the software Geneious version 5.6.4 created by Biomatters, available from www.geneious.com.

SIVmac239 encodes two polypeptides in ARFs of Env that are frequent targets of CTL responses in SIV-infected animals (termed ARF-1 and ARF-10) [21]. Here, we report a comprehensive examination of the kinetics of viral escape from CTL responses directed against these two novel polypeptides. To examine viral escape in ARFs -1 and -10 in acute SIV infection, we designed a PCR amplicon spanning both of these ARFs, in addition to the first 87 amino acids of the overlapping Env protein (Figure 1). This amplicon allowed us to assess selection pressure exerted by anti-SIV CTL in three overlapping open reading frames (ORFs).

## Materials and Methods

### Ethics Statement

All samples used in this study were from animals used for other studies and were collected and cryopreserved as part of routine sample banking for those studies. Hence, no animals were infected with SIV, euthanized or sampled in any way specifically for this study. The animals used in this study were rhesus macaques (*Macaca mulatta*) and were part of the breeding colony at the Wisconsin National Primate Research Center. All animals were cared for in accordance with the guidelines prescribed by the NIH Guide to Laboratory Animal Care and in accordance with the recommendations of the Weatherall report – “The use of non-human primates in research”. All work conducted on the animals used in this study was performed under protocols approved by the University of Wisconsin Graduate School Institutional Animal Care and Use Committee (protocol numbers are as follows; g00559 for r04028 and rh2261, g00473 for r97035 and g00409 for r97111). As per approved protocols, all animals were euthanized with an IV overdose (greater than or equal to 50 mg/kg or to effect) of sodium pentobarbital or equivalent, preceded by ketamine (at least 15 mg/kg body weight, IM). Qualified veterinarians and technicians performed necropsies for tissue collection.

### SIV sample preparation, PCR amplification and sequencing

Cell-free plasma was obtained from EDTA anticoagulated whole blood by using Ficoll-Paque Plus (GE Healthcare Bioscience) and density centrifugation. Plasma viral RNA was isolated as previously described [25]. Viral RNA was reverse transcribed and amplified by using the SuperScript III One-Step RT-PCR kit [(with high fidelity taq (Invitrogen)], using primers listed in Table 1. The reverse transcription-PCR conditions were as follows: 50°C for 30 min; 95°C for 15 min; 45 cycles of 98°C for 1 min, 58°C for 1 min, and 72°C for 2.5 min; and 68°C for 20 min. Following RT-PCR, samples were gel purified using QIAGEN MinElute columns (Qiagen, Valencia, CA). Samples were quantified using Quant-IT HS reagents (Invitrogen, Carlsbad, California).

**Table 1:** MID-tagged oligonucleotides used to generate ARF1/ARF10/Env specific amplicons.

Primer Name	Primer Sequence		
	*Roche Adaptor*	*MID tag*	*Seq-specific primer*
5_ARF_MID12	CGTATCGCCTCCCTCGCGCCATCAG	***TACTGAGCTA***	TGCTACCATTGCCAGTTTTG
3_ARF_MID12	CTATGCGCCTTGCCAGCCCGCTCAG	***TACTGAGCTA***	TCCTCTATTGCCTGTTCTGTGA
5_ARF_MID13	CGTATCGCCTCCCTCGCGCCATCAG	***CATAGTAGTG***	TGCTACCATTGCCAGTTTTG
3_ARF_MID13	CTATGCGCCTTGCCAGCCCGCTCAG	***CATAGTAGTG***	TCCTCTATTGCCTGTTCTGTGA
5_ARF_MID18	CGTATCGCCTCCCTCGCGCCATCAG	***TCTACGTAGC***	TGCTACCATTGCCAGTTTTG
3_ARF_MID18	CTATGCGCCTTGCCAGCCCGCTCAG	***TCTACGTAGC***	TCCTCTATTGCCTGTTCTGTGA
5_ARF_MID19	CGTATCGCCTCCCTCGCGCCATCAG	***TGTACTACTC***	TGCTACCATTGCCAGTTTTG
3_ARF_MID19	CTATGCGCCTTGCCAGCCCGCTCAG	***TGTACTACTC***	TCCTCTATTGCCTGTTCTGTGA
5_ARF_MID25	CGTATCGCCTCCCTCGCGCCATCAG	***TCGTCGCTCG***	TGCTACCATTGCCAGTTTTG
3_ARF_MID25	CTATGCGCCTTGCCAGCCCGCTCAG	***TCGTCGCTCG***	TCCTCTATTGCCTGTTCTGTGA
5_ARF_MID26	CGTATCGCCTCCCTCGCGCCATCAG	***ACATACGCGT***	TGCTACCATTGCCAGTTTTG
3_ARF_MID26	CTATGCGCCTTGCCAGCCCGCTCAG	***ACATACGCGT***	TCCTCTATTGCCTGTTCTGTGA
5_ARF_MID27	CGTATCGCCTCCCTCGCGCCATCAG	***ACGCGAGTAT***	TGCTACCATTGCCAGTTTTG
3_ARF_MID27	CTATGCGCCTTGCCAGCCCGCTCAG	***ACGCGAGTAT***	TCCTCTATTGCCTGTTCTGTGA
5_ARF_MID28	CGTATCGCCTCCCTCGCGCCATCAG	***ACTACTATGT***	TGCTACCATTGCCAGTTTTG
3_ARF_MID28	CTATGCGCCTTGCCAGCCCGCTCAG	***ACTACTATGT***	TCCTCTATTGCCTGTTCTGTGA
5_ARF_MID29	CGTATCGCCTCCCTCGCGCCATCAG	***ACTGTACAGT***	TGCTACCATTGCCAGTTTTG
3_ARF_MID29	CTATGCGCCTTGCCAGCCCGCTCAG	***ACTGTACAGT***	TCCTCTATTGCCTGTTCTGTGA
5_ARF_MID30	CGTATCGCCTCCCTCGCGCCATCAG	***AGACTATACT***	TGCTACCATTGCCAGTTTTG
3_ARF_MID30	CTATGCGCCTTGCCAGCCCGCTCAG	***AGACTATACT***	TCCTCTATTGCCTGTTCTGTGA
5_ARF_MID31	CGTATCGCCTCCCTCGCGCCATCAG	***AGCGTCGTCT***	TGCTACCATTGCCAGTTTTG
3_ARF_MID31	CTATGCGCCTTGCCAGCCCGCTCAG	***AGCGTCGTCT***	TCCTCTATTGCCTGTTCTGTGA
5_ARF_MID32	CGTATCGCCTCCCTCGCGCCATCAG	***AGTACGCTAT***	TGCTACCATTGCCAGTTTTG
3_ARF_MID32	CTATGCGCCTTGCCAGCCCGCTCAG	***AGTACGCTAT***	TCCTCTATTGCCTGTTCTGTGA
5_ARF_MID33	CGTATCGCCTCCCTCGCGCCATCAG	***ATAGAGTACT***	TGCTACCATTGCCAGTTTTG
3_ARF_MID33	CTATGCGCCTTGCCAGCCCGCTCAG	***ATAGAGTACT***	TCCTCTATTGCCTGTTCTGTGA
5_ARF_MID34	CGTATCGCCTCCCTCGCGCCATCAG	***CACGCTACGT***	TGCTACCATTGCCAGTTTTG
3_ARF_MID34	CTATGCGCCTTGCCAGCCCGCTCAG	***CACGCTACGT***	TCCTCTATTGCCTGTTCTGTGA
5_ARF_MID35	CGTATCGCCTCCCTCGCGCCATCAG	***CAGTAGACGT***	TGCTACCATTGCCAGTTTTG
3_ARF_MID35	CTATGCGCCTTGCCAGCCCGCTCAG	***CAGTAGACGT***	TCCTCTATTGCCTGTTCTGTGA
5_ARF_MID36	CGTATCGCCTCCCTCGCGCCATCAG	***CGACGTGACT***	TGCTACCATTGCCAGTTTTG
3_ARF_MID36	CTATGCGCCTTGCCAGCCCGCTCAG	***CGACGTGACT***	TCCTCTATTGCCTGTTCTGTGA
5_ARF_MID37	CGTATCGCCTCCCTCGCGCCATCAG	***TACACACACT***	TGCTACCATTGCCAGTTTTG
3_ARF_MID37	CTATGCGCCTTGCCAGCCCGCTCAG	***TACACACACT***	TCCTCTATTGCCTGTTCTGTGA
5_ARF_MID38	CGTATCGCCTCCCTCGCGCCATCAG	***TACACGTGAT***	TGCTACCATTGCCAGTTTTG
3_ARF_MID38	CTATGCGCCTTGCCAGCCCGCTCAG	***TACACGTGAT***	TCCTCTATTGCCTGTTCTGTGA
5_ARF_MID39	CGTATCGCCTCCCTCGCGCCATCAG	***TACAGATCGT***	TGCTACCATTGCCAGTTTTG
3_ARF_MID39	CTATGCGCCTTGCCAGCCCGCTCAG	***TACAGATCGT***	TCCTCTATTGCCTGTTCTGTGA
5_ARF_MID40	CGTATCGCCTCCCTCGCGCCATCAG	***TACGCTGTCT***	TGCTACCATTGCCAGTTTTG
3_ARF_MID40	CTATGCGCCTTGCCAGCCCGCTCAG	***TACGCTGTCT***	TCCTCTATTGCCTGTTCTGTGA

MID tags are in bold italics. Sequence-specific primer is 3′ to the MID tag while adaptor sequence is 5′ to the tag in each oligonucleotide. The number of MID-tagged primers is less than the number of samples as some primers were re-used in separate runs.

Pyrosequencing was performed on a Roche Genome Sequencer Junior instrument. Sequence files (SFF) generated following the sequencing runs were analyzed in two ways; with Geneious software version 5.6.6, created by Biomatters, available at www.geneious.com as well as a custom analysis pipeline, previously described [12], [26], [27] with highly similar results. The custom pipeline utilized Samtools [28] and BioPerl [29]. This pipeline has been made available as a module for LabKey Server, an open-source platform for the management of scientific data [30] (www.labkey.com). The LabKey SequenceAnalysis module provides a web-based interface to initiate analyses, manage data, and view results. The source code behind this pipeline is available in a subversion repository (https://hedgehog.fhcrc.org/tor/stedi/trunk/server/customModules/SequenceAnalysis). The pipeline performs three steps: pre-processing of sequence, alignment, and SNP calling. The raw sequence data was processed as follows: sequences were converted to FASTQ format, separated by MID tag, trimmed using quality scores. Next, the sequences were aligned to SIVmac239 (Genbank ID: M33262.1) using BWA-SW [31]. All SNPs were evaluated and low-quality SNPs discarded. Importantly, the identity of the associated read was retained for each SNP, which allows the phase of SNPs to be considered in translation and reconstruction of haplotypes across CTL epitopes. The information produced during the analysis process was stored in the LabKey database, from which results were viewed and reports generated.

As controls, we also sequenced this region of the viral genome from a wild type viral stock of SIVmac239 as well as the SIV plasmid DNA used for the initial production of virus for inoculations. No mutations were detected that exceeded our 1% threshold for analysis (data not shown), validating both our threshold and the statistical analyses indicating CTL-mediated selection pressure. This threshold is considered conservative and useful for studies identifying specific mutations with biological importance, such as CTL escape mutations [32]. In many cases, multiple nucleotide changes underlie a given amino acid change. This observation is critical for determining which reading frame translation is the target of selection. However, in nearly all cases, all underlying nucleotide changes were non-synonymous in the same reading frames, resulting in almost no ambiguities.

For Sanger sequencing experiments we reverse transcribed and amplified SIV viral RNA in the same manner as used for pyrosequencing and using the following primers (Forward; GGAGGAAATCCTCTCTCAGC, and Reverse; ACCAAATCTGCAGAGTACCA). Sequences were performed in duplicate using Big Dye Terminator V3.1 on an Applied Biosystems 3130 Genetic Analyzer and purified using an Edge Biosystems PERFORMA Ultra 96 well plate. Sequences were trimmed and analyzed using Geneious software version 5.6.4, created by Biomatters, available at www.geneious.com.

### Statistical analysis of viral escape

Sequence analyses were based on data sets from which we excluded all haplotypes (i.e., individual sequences) represented by a single read in a given sample (i.e., sequences sampled from a given host monkey at a given time). Preliminary analyses showed that haplotypes occurring only once were more likely to be short relative to other reads or to align poorly. In some samples, certain other haplotypes that occurred two (or in one case three) times in the sample were also excluded because they aligned poorly with the majority of reads. Sequences were aligned with the inoculum sequence using CLUSTAL X [33]. The number of synonymous substitutions per synonymous site (*d_S_*) and of nonsynonymous substitutions per nonsynonymous (*d_N_*) site between each sequence and the inoculum sequence were estimated by Nei and Gojobori's method [34].

### ELISPOT and epitope mapping

ELISPOTS were performed as previously described [35] using peptides 15 amino acids in length, overlapping by 11 amino acids. Epitope mapping using IFN–γ ELISPOT was conducted using log dilutions of peptides in ELISPOT plates using either 100,000 PBMC per well (in the case of ex vivo epitope mapping) or 20,000 T cells per well (in the case of r04028 where antigen-specific T cell lines were used for epitope mapping). Peptides used for mapping were 15, 11, 10, 9 or 8 amino acids in length. We used dilutions of peptides in duplicate wells to identify the shortest peptides that elicited the highest cytokine production across the broadest range of dilutions; these peptides were designated the minimal optimal epitopes.

### CTL lines and intracellular cytokine staining

Antigen-specific CTL lines against the AF8 epitope were created by exposing PBMC from r04028 from 12 weeks post infection to irradiated, autologous BLCL pulsed for 2 hours with a 15-mer peptide (WESAAYRHLAFKCLW) from ARF-10 that contains the AF8 epitope within at a concentration of 10 uM. After 2 hours, the cells were washed of free peptide and added to 5 million PBMC and cultured in RPMI-1640 media (HyClone) supplemented with 15 percent fetal bovine sera (FBS, Lonza) and 50 U/ml IL-2 [36].

Peptide stimulation was repeated weekly for 4 weeks and the cells were used in assays.

Intracellular cytokine staining (ICS) was performed by combining 100,000 cells from the CTL lines with 100,000 autologous BLCL, along with peptide and GolgiPlug (brefeldin A – BD Biosciences). Cells stimulated with Concanavalin A (Sigma) were used as positive controls for the assay and unstimulated cells were used as negative controls. Cells were incubated for 18 hours after which they were stained for 30 minutes with antibodies against CD8 (APC-Cy7, clone SK1, BD Biosciences) and CD3 (PE-Cy7, clone SP34-2, BD Biosciences). Cells were fixed using stabilizing fixative (BD Biosciences). At least 15 minutes after fixing, cells were washed once with PBS buffer containing 10% FBS. Cells were permeabilized using Perm/Wash reagent (BD Biosciences) and antibodies against IFN-γ (PerCP-Cy5.5, clone 4S.B3, BD Biosciences) and TNF-α (APC, clone Mab11, BD Biosciences) were added and incubated for 45 minutes. At the completion of the incubation, the cells were washed twice with Perm/Wash reagent. Finally, cells were fixed with stabilizing fixative and data was acquired using a BD Fortessa instrument. Data was analyzed using Flowjo software, version 10.0.4.

## Results and Discussion

Acute phase viral escape from CTL responses is a universal feature of AIDS virus infection, both in SIV-infected rhesus macaques and HIV-infected humans [37]–[39]. Descriptions of viral escape from cryptic epitope-specific CTL responses are rare and little is known about whether CTL target cryptic epitopes in the first several weeks of infection and whether these responses drive viral evolution. We initially showed that SIV escapes CTL responses that target a cryptic epitope restricted by Mamu-B*017:01 in Indian Rhesus macaques, the expression of which is correlated with extraordinary control of SIV replication [18]. Subsequent reports demonstrated that HIV likewise evolves to evade cryptic epitope-specific CTL responses [17], [19], [20]. However, few studies have shown that CTL responses targeting cryptic epitopes within the critical first few weeks of AIDS virus infection can exert sufficient selective pressure on AIDS viruses to drive viral evolution. This report fills that gap and underscores the importance of these responses to the total AIDS virus-specific CTL response in vivo.

ARF-1 is putatively a 69 amino acid polypeptide starting with the first AUG codon and ending with a stop codon and translated from the +1 reading frame relative to the Env-encoding ORF. We previously published two epitopes in ARF-1, one of which was targeted early in infection [21]. ARF-10 is putatively a 73 amino acid polypeptide translated from the Env-encoding mRNA, via translation initiation at the Rev AUG start codon and continued translation into the intron [40]. For the present and previous study [21], we tested for CTL responses against these polypeptides using overlapping peptides (15-mers overlapping by 11) that encompass the predicted translated portions of the ARFs. For ARF-10, these peptides included the last several amino acids of the first exon of Rev to ensure identification of responses that target epitopes overlapping the exon/intron boundary. We used these peptides in IFN–γ ELISPOT assays to identify T cell responses targeting this polypeptide in Rhesus macaques infected with SIVmac239 (or experimental mutants of SIVmac239) as part of previous studies. Identified responses were mapped to the minimal optimal epitope using serial dilutions of peptides and either PBMC from the responding animal or antigen-specific T cell lines grown against the targeted 15-mer (from r04028, explained in detail in the Results and Discussion and Materials and Methods sections).

In this study, we sought not only to identify epitopes targeted in ARFs -1 and -10 during the acute phase of infection but also to measure CTL-mediated selection pressure on these epitopes. Sequence evolution was identified using deep pyrosequencing of a small, highly targeted PCR amplicon that spanned ARFs -1 and -10, including the first exon of Rev and the first portion of the Env protein, which overlaps both ARFs. The genomic locations of ARFs -1 and -10 and the forward and reverse primers used to amplify the region from SIV are depicted in figure 1. The primers used, including Roche adaptor, multiplex identifier (MID) and sequence-specific primer are depicted in table 1. Finally, we conducted *d_N_/d_S_* analyses and compared the kinetics of the accumulation of non-synonymous intra- vs. extra-epitopic changes in ARF-1 and ARF-10 in order to measure CTL-induced selection on the epitopes.

**Figure 2:**
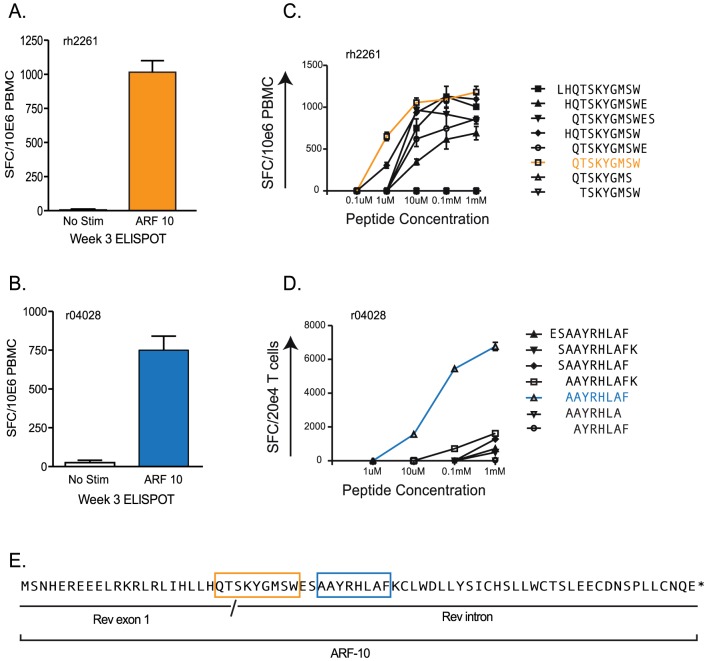
Acute phase CTL responses against ARF-10 in two SIV-infected Rhesus macaques. A) and B) Magnitude of CTL responses against 15-mers in ARF-10 in Rhesus macaques rh2261, A, and r04028, B, as measured by IFN–γ ELISPOT using cryopreserved PBMC harvested 3 weeks after initial SIV infection. The SIV infection history of the animals is described in the text. Bars are color coded to match with remaining panels in the figure. C) and D) Peptide dilutions to fine map the minimal optimal epitopes targeted in ARF-10 by rh2261, C, and r04028, D. For rh2261, we used PBMC harvested in the acute phase of infection. For animal r04028, due to PBMC sample limitations, we expanded antigen-specific CTL by exposing PBMC to autologous irradiated BLCL pulsed with the responsive 15-mer for several weeks and used these cells in an ELISPOT. For panels A–C, we used 100,000 PBMC per well, in duplicate, in ELISPOT plates. For the epitope mapping with r04028, we used 20,000 antigen-specific cells per well combined with 10,000 autologous BLCL per well as antigen presenting cells. Peptides tested are shown at right of each mapping panel and the peptides we determined represented the minimal epitopes are shown in color to match panels A and B. E) The mapped epitopes within ARF-10 are boxed using the color scheme described above.

Animals r04028 and rh2261 were recently infected with SIVmac239 experimentally mutated to harbor “pre-escaped” epitopes typically presented by Mamu-B*008:01 as part of an unrelated previous study [41]. Three weeks after infection, we used IFN—γ ELISPOT to enumerate SIV-specific T cell responses targeting ARFs -1 and -10. Both animals responded to overlapping peptides in ARF-10 at magnitudes near 1,000 spot forming cells (SFC) per million PBMC (Figure 2A and 2B). We next used a panel of peptides ranging in length from 8 to 11 amino acids spanning the targeted 15-mers to map the responses to the minimal optimal epitopes. We used serial dilutions of these peptides and cryopreserved PBMC from animal rh2261 in IFN–γ ELISPOT assays to map the targeted epitope to the QW9 epitope (Figure 2C). PBMC from animal r04028 were not available from an acute phase time point and later time points showed no or very little response (data not shown). Therefore, to map the targeted epitope, we expanded antigen-specific CTL from this animal by exposing PBMC from 12 weeks post infection to irradiated autologous BLCL pulsed with the 15-mer peptide against which the response was originally detected (WESAAYRHLAFKCLW). After weekly stimulation for four weeks, we used this cell line in ELISPOT assays, similar to rh2261, and identified the minimal optimal epitope in this animal as the AF8 epitope (figure 2D). In the absence of PBMC from an acute time point, the use of a CTL line expanded against the parent 15-mer peptide for a minimum number of in vitro stimulations was the least biased approach to mapping this epitope. The locations of the mapped epitopes within ARF-10 are show in figure 2E.

**Figure 3:**
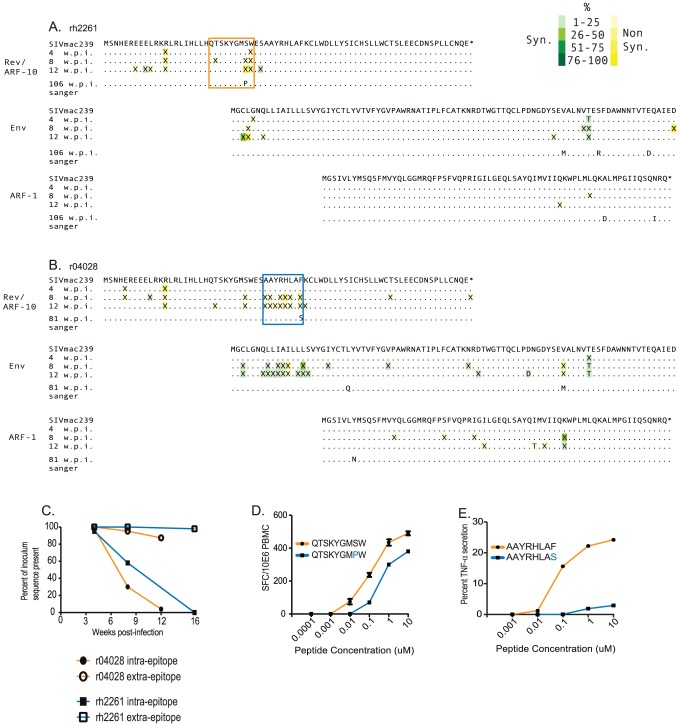
Viral sequence evolution in overlapping reading frames encoding Env and ARFs -1 and -10. We used next generation pyrosequencing (454 Life Sciences) of the amplicon depicted in figure 1 to sequence the portion of SIV encoding ARF-10, ARF-1 and the first 89 amino acids of Env. A) Kinetics of viral evolution at weeks 4, 8 and 12 from animal rh2261. Mutations synonymous in a given reading frame are boxed in green and non-synonymous changes are boxed in yellow. Matching residues are depicted as “.”. Mutations are depicted as “X” when nucleotide mutations in a given codon could give rise to more than one amino acid. The shade of the box surrounding a mutation represents the frequency of underlying mutations at that codon. The key is in the upper right of the figure. Only mutations present at >1% are shown. The minimal epitope mapped in figure 2 is shown boxed in the same animal-specific color coding as in figure 2; The QW9 epitope for animal rh2261, A, and the AF8 epitope for animal r04028, B. We used simple Sanger sequencing of virus derived from each animal at their individual times of euthanasia to determine the consensus escape patterns in this region after the resolution of the acute phase of infection. C) The frequency of amino acid sequences representing the inoculum within the targeted epitope (intra-epitope, solid shapes) and outside the targeted epitopes (extra-epitope, open shapes) within ARF-10 plotted against weeks post infection. D) IFN-γ ELISPOT assay of recognition of the peptide representing the wild type QW9 sequence (orange) versus the escape variant (orange) by PBMC isolated from animal rh2261 from 4 weeks post infection. E) Intracellular cytokine staining (ICS) assay to measure recognition of a CTL line from r04028 against the wild type AF8 peptide (orange) versus the escape variant (blue). Details of the CTL line are described in the text.

We next wished to determine if the CTL targeting ARF-10 were sufficiently potent to select for viral escape mutations in vivo. We used primers from Table 1 to reverse transcribe and amplify replicating SIV isolated from cell-free plasma from four, eight and 12 weeks post-infection in animal r04028 and weeks four, ten and 16 in animal rh2261. We used 454 Life Sciences (Roche) deep pyrosequencing of these amplicons and found clusters of non-synonymous mutations within the targeted epitopes by eight weeks post-infection in virus isolated from both animals (Figure 3A and 3B). Viral escape from CTL responses is generally characterized by small numbers of amino acid substitutions (often just one) within a restricted epitope that either reduces binding of the virus-derived peptide to the MHC-I molecule or reduces the affinity of the T cell receptor (TCR) on the responding CTL for the MHC-I: peptide complex. Alternately, mutations can arise outside the epitope that reduce the efficiency with which the epitope itself is liberated from the parent polypeptide by cellular proteasomes and immunoproteasomes. Our pyrosequencing data was restricted to acute time points soon after the initial CTL responses arose and do not demonstrate which mutations resulted in stable escape. Therefore, we next used Sanger sequencing to sequence this portion of the circulating virus from plasma samples taken at the time of euthanasia (81 weeks post infection for r04028 and 106 weeks post infection in rh2261) to determine if viral evolution had coalesced onto a single dominant mutation. In both animals a single amino acid change was identified within the epitopes, likely representing the stable escape mutation.

The clear clustering of non-synonymous mutations within the targeted epitopes, particularly the AF8 epitope targeted by r04028, strongly suggests archetypal viral escape from a CTL response. *d_N_/d_S_* analysis supported this suggestion (p<0.05). Indeed, in both animals, the dramatic drop in the portion of sequence reads matching the inoculum within the targeted epitopes during the acute phase (Figure 3C, solid shapes) compared to the relative constancy of the extra-epitopic portion of ARF-10 is strongly suggestive of viral escape from these CTL responses in vivo.

To test if these mutations detected late in infection reduced or eliminated CTL recognition, we thawed cryopreserved PBMC from animal rh2261 from four weeks post infection and used IFN-γ ELISPOT to measure recognition of serial dilutions of the wild type QW9 peptide versus QW9 harboring a position 8, S to P mutation. Recognition of the mutated peptide was reduced significantly as compared to wild type. However, it was not eliminated (figure 3D). This data provides further evidence that the mutation was the result of selection by antiviral CTL. It is curious that selection did not favor a mutant in an MHC anchor residue, assuming the virus could do so without fitness consequences. It is possible that this intra-epitope mutation impairs processing of the MHC-bound 9-mer, which would not be evidenced by recognition of peptides but would require introduction of the mutation into the virus and measurement of recognition of infected cells. This situation was described for a cryptic epitope in HIV-1 [17]. As mentioned above, acute phase PBMC were no longer available from animal r04028 that could be used to compare recognition of the wild type AF8 peptide with the mutated peptide harboring a position 8 F to S mutation (the intra-epitope mutation detected at time of euthanasia). To remedy this, we used an antigen specific T cell line expanded against the 15-mer harboring the AF8 epitope (WESAAYRHLAFKCLW). We found this cell line to have high background for IFN-γ so we instead used intracellular cytokine staining and measured its ability to produce TNF-α in response to serial dilutions of the wild type and the mutant peptide. This cell line responded well to several dilutions of the wild type peptide but very poorly to the mutant peptide (figure 3E). This is not surprising, given the mutation is in a presumed anchor residue (position 9 of 9).

We note two important observations in our evolutionary analysis of CTL recognition of ARF-10. First, in virus circulating in animal r04028, nearly all mutations within the targeted AF8 epitope that exceeded our threshold of 1% were synonymous in the overlapping Env-encoding ORF, presumably indicating that this short stretch of Env was under pressure to maintain its amino acid sequence. Finally, we note that viral evolution in the QW9 epitope targeted by rh2261 was entirely clustered in the carboxyl end of the epitope. We hypothesize that selection might have favored variants that escaped the CTL response while maintaining the amino acid sequence of the first exon of Rev and the RNA sequence surrounding the Rev splice donor site, the disruption of which would likely incur a substantial cost to viral fitness.

**Figure 4:**
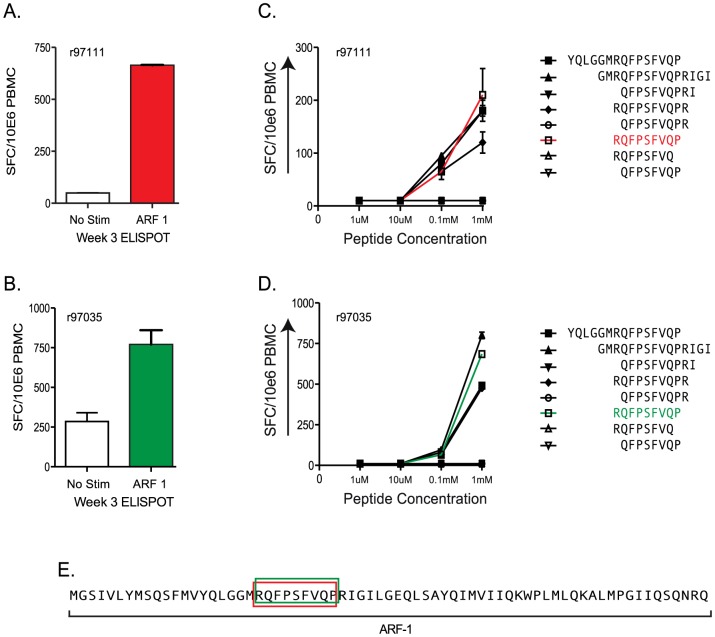
Acute phase CTL responses against ARF-1 in two SIV-infected Rhesus macaques. A) and B) Similar to panels A and B in figure 2. Here, we depict the week 3 responses to 15-mers within ARF-1 in two animals, r97111, A, and r97035. The infection history of these animals is described in the text. C) and D) The minimal epitope was mapped to the RP9 peptide in both animals using serial 10-fold dilutions of peptide in IFN–γ ELISPOT assays. The peptides used are shown at right and the RP9 peptide, which was determined to be the minimal epitope is shown in orange. E) The location of the RP9 epitope within ARF-1.

In our previous study [21], we showed that several animals made high-frequency CTL responses against the RP9 epitope in ARF-1. In this report, we show a comprehensive examination of the CTL responses against this epitope in two animals and the kinetics of the subsequent viral escape from this response. Animal r97035 was infected with SIVmac239 engineered to harbor escape mutations in epitopes in Gag, Tat and Nef [42], [43]. This animal was a long-term non-progressor and survived with low but detectable viremia for more than four years after initial SIV infection. Animal r97111 was infected with wild type SIVmac239 as part of an unpublished previous study. Each animal responded to overlapping 15-mer peptides in ARF-1 (figure 4A and 4B). The responses were fine-mapped to the same RP9 epitope using serial 10-fold dilutions of peptides representing truncations of the targeted 15-mer in IFN–γ ELISPOT assays (figure 4C and 4D). The position of the RP9 epitope within the ARF-1 polypeptide is shown in figure 4E. In both animals, nearly identical functional avidities are shown between one of the targeted 15-mers and the RP9 peptide. Presumably, this is due to highly efficient liberation of the RP9 9-mer from this peptide for unknown reasons. PBMC were not available from the same acute time points in which the responses were first detected and, hence, the magnitudes of the responses in both animals are lower in the epitope mapping ELISPOTS than they are in the initial ELISPOTS.

**Figure 5:**
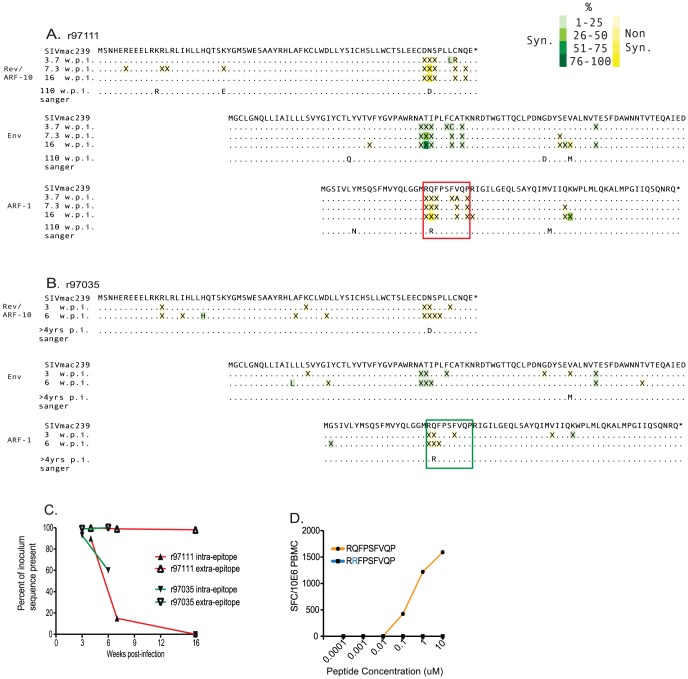
Viral sequence evolution in overlapping reading frames encoding Env and ARFs -1 and -10. Sequence evolution in overlapping reading frames in two animals that targeted ARF-1 at three weeks post-infection; r97111, A, and r97035, B. The targeted epitope is shown boxed in orange for each animal. Data from acute phase time points are from deep pyrosequencing, while the single chronic time point assayed in each animal was sequenced with Sanger sequencing. The key is in the upper right and is identical to that used in figure 3 and details of sequence methods and analysis are depicted in the caption for figure 3 and in the Materials and Methods section. C) The portion of sequence reads matching inoculum within targeted epitopes (solid shapes) contrasted with the portion matching inoculum outside the epitope in ARF-1. D) IFN-γ ELISPOT assay to measure the ability of PBMC from r97111 to recognize dilutions of the wild type RP9 peptide (orange) versus escape mutant (blue) peptide.

We next tested whether selection favored variants of RP9 that evaded the RP9-specific CTL response. To accomplish this, we used the same focused pyrosequencing approach we used with ARF-10. Specifically, we sequenced virus extracted at acute phase time points to determine whether viral escape occurs in this epitope and how long after infection escape becomes detectable. SIV from both animals harbored clusters of mutations in this epitope as early as 3 and 3.7 weeks post infection. By 16 weeks post-infection in r97111, there was a clear pattern favoring the position two Q to R mutation. Indeed, when we used Sanger sequencing at time points much later in infection (110 weeks p.i. in r97111 and more than 4 years p.i. in r97035), we detected the same single mutation in both animals at position two of the epitope (figure 5A and 5B). Position two of an MHC-I restricted epitope is nearly always important for anchoring peptides to the MHC-I molecules. Hence, this escape pattern would indicate that the virus evolves in convergent fashion in these animals to escape the RP9-specific CTL response. Patterns of acute viral escape in these animals was similar to that in the AF8 epitope in r04028 in that a cluster of mutations appears early in infection that is almost entirely synonymous in the overlapping Env ORF. We interpret these data to mean that there is pressure to maintain the Env amino acid sequence in this small region. It should also be noted that the patterns substitutions could suggest immune targeting of the region of ARF-10 overlapping the RP9 epitope within ARF-1. However, we did not detect T cell activity against ARF-10 in these animals (data not shown).

Finally, we assessed CTL-induced selection in the RP9 epitope in SIV from each animal. As with the ARF-10 restricted epitopes, *d_N_/d_S_* analysis was significant (p<0.05) and we also observed dramatic accumulations of non-synonymous mutations within the epitope, represented by loss of inoculum sequence (figure 5C, closed symbols). In contrast, the extra-epitopic portions of ARF-1 were largely maintained as wild type through acute phase sampling (figure 5C, open symbols), and even at much later time points as measured by Sanger sequencing. We next thawed acute phase (week 4 post infection) PBMC from animal r97111 to compare recognition of the wild type RP9 epitope to the mutant peptide. We detected strong recognition of the wild type peptide over several orders of magnitude of peptide concentrations but no recognition of the mutant peptide (figure 5D). Animal r97035 was infected several years prior to this study and acute phase CTL were no longer available to complete a comparable experiment. However, since the responses mapped to the same minimal epitope and viral evolution was highly convergent in each animal, these data provided strong evidence that the virus evolved as a direct result of the CTL targeting the RP9 epitope in both animals.

The challenge viruses used to inoculate several of the animals used in this study contained mutations in established T cell epitopes in order to test hypotheses distinct from those tested here. It is unknown whether this affected the kinetics of the ARF-1 and ARF-10 specific CTL responses and subsequent viral escape. In the case of ARF-1, this appears not to be the case. Animal r97035 was infected with a version of SIVmac239 containing escape mutations in three epitopes [42], while animal r97111 was infected with wild type SIVmac239, and the kinetics of the ARF-1 RP9 targeted CTL response and viral escape were nearly identical in both animals. Hence, cryptic epitope-specific CTL responses are not inherently subdominant. The RP9-specific CTL response appears to be an immunodominant acute phase CTL response, irrespective of epitope sequences in the challenge virus outside this epitope.

Taken together, our data substantiate an important role for CTL against cryptic epitopes during AIDS virus infection. Viral escape is an important measure of *in vivo* CTL efficacy. Hence, CTL against cryptic epitopes could be important components of the total AIDS virus-specific responses and their role in vaccine modalities should be investigated. Our data also suggest that ARFs -1 and -10 appear to be translated as a normal function of SIV replication, despite being favorite targets of host T cell responses. It may prove informative to investigate the biochemical properties of these novel polypeptides in greater detail.
